# Gold Nanotheranostics: Photothermal Therapy and Imaging of Mucin 7 Conjugated Antibody Nanoparticles for Urothelial Cancer

**DOI:** 10.1155/2015/813632

**Published:** 2015-03-05

**Authors:** Chieh Hsiao Chen, Yi-Jhen Wu, Jia-Jin Chen

**Affiliations:** ^1^Institute of Biomedical Engineering, National Cheng Kung University, 1 University Road, Tainan 701, Taiwan; ^2^Department of Urology, China Medical University Beigang Hospital, 123 Sin-der Road, Beigang, Yunlin 651, Taiwan

## Abstract

*Objective*. To kill urothelial cancer cells while preserving healthy cells, this study used photothermal therapy (PTT). PTT techniques target urothelial cancer cells using gold nanoparticles (GNPs) and a green light laser. *Materials and Methods*. The GNPs were conjugated with anti-Mucin 7 antibodies, which acted as a probe for targeting tumor cells. Conjugated GNPs were exposed to a green light laser (532 nm) with sufficient thermal energy to kill the transitional cell carcinomas (TCCs). *Results*. According to our results, nanoparticles conjugated with Mucin 7 antibodies damaged all types of cancer cells (MBT2, T24, 9202, and 8301) at relatively low energy levels (i.e., 500 laser shots at 10 W/cm^2^ in power, 1.6 Hz in frequency, and 300 ms in duration). Nonconjugated nanoparticles required 30 W/cm^2^ or more to achieve the same effect. Cell damage was directly related to irradiation time and applied laser energy. *Conclusions*. The minimally invasive PTT procedure combined with Mucin 7 targeted GNPs is able to kill cancer cells and preserve healthy cells. The success of this treatment technique can likely be attributed to the lower amount of energy required to kill targeted cancer cells compared with that required to kill nontargeted cancer cells. Our in vitro pilot study yielded promising results; however, additional animal studies are required to confirm these findings.

## 1. Introduction

Urothelial cancer accounts for approximately 90% of urinary bladder cancers. Other types of bladder cancers include adenocarcinoma (2%), squamous cell carcinoma (5–10%), and other rare carcinomas (e.g., carcinosarcoma, undifferentiated carcinoma, etc.). Intravesical therapy is a selective adjuvant therapy used to treat nonmuscle invasive urothelial cancer. Intravesical therapies include bacillus Calmette-Guérin (BCG), mitomycin, and epirubicin. Among the active agents used in these treatments, BCG is considered the most effective [1]. Despite the beneficial effects of these treatment regimes, cancer recurrence rates are still as high as 40% in BCG users and approximately 53% in the recipients of other chemotherapy agents [2, 3]. The side effects associated with the intravesical instillation of BCG include cystitis (67%), haematuria (23%), fever (25%), and urinary frequency (71%) [4]. Researchers have not yet established an effective means of directly targeting bladder cancers early in the recurrence stage. Addressing this key issue is crucial to improving disease prognosis and the quality of life among patients.

Various hyperthermia techniques such as laser, focused ultrasound, and microwave have been used to destroy target cells; however, simple heating techniques can damage normal tissue. To avoid heat damage, photothermal therapy (PTT) techniques combining the use of gold nanoparticles (GNPs) for the targeting of antibodies have been developed. In this type of cancer treatment, conjugated antibodies target cancer cells, allowing GNPs to attack tumors with a high degree of precision. This targeting action is even successful when the tumor is too small to be detected by other imaging techniques. Due to the specificity of PTT techniques, the healthy surrounding tissues are rarely damaged.

Due to developments in surface plasmon resonance (SPR) technology, the efficacy of PTT/GNP techniques in cancer treatment has generated a great deal of interest in recent years [5, 6]. This study used a 532 nm laser system to excite the GNPs. This light-heat energy transformation is highly efficient making it possible for PTT to minimize damage to normal tissue, decrease the adverse side effects of treatment, and shorten the course of treatment.

The use of GNPs in cancer diagnosis and therapy has been widely investigated [5, 7] because these particles are known to have excellent SPR characteristics and strong biocompatibility. GNPs have been used in radiology since the 1950s. Compared with other kinds of nanoparticles, such as the silica used in the core of core-shell nanoparticles, magnetic nanoparticles [8, 9], and quantum dots [10], GNPs are relatively nontoxic [5]. Other obvious advantages of GNPs include chemical stability and high affinities to biomolecules [11, 12]. Furthermore, GNP can be used in conjunction with other agents for cancer detection and therapy. These particles demonstrate great potential and versatility in clinical applications.

Gold nanorods and gold/silica nanoshells have recently been used as imaging and therapeutic agents in the treatment of cancers, such as breast cancer [13–16], brain cancer [17], oral cavity cancer [5], and prostate cancer. Interest in these materials can be attributed to the similar absorption wavelength they share with near infrared (NIR) rays [18–20]. Spherical gold nanoparticles 50 nm in diameter have the best capacity for uptake by mammalian cells (i.e., compared with nanoparticles 14 nm, 30 nm, 74 nm, and 100 nm in size, as well as with rod-shaped nanoparticles 40 nm × 14 nm and 74 nm × 14 nm) [21]. Destroying tumor cells and preserving normal cells require a targeting system and monoclonal antibodies to improve the targeting ability of the nanoparticles. Targets are selected according to the expression of antigens in cancers. Presently, EGFR, Mucin 7, and cytokeratin 20 are commonly used to target bladder cancer [22–24]. This study selected Mucin 7 as the target to determine whether urothelial cancers can be destroyed by PTT with immunized GNP.

## 2. Materials and Methods

### 2.1. Preparation of Gold Nanospheres

GNPs with an average size of 47 nm were synthesized through chemical reduction in accordance with the method proposed by Kimling et al. [25]. Gold chloride trihydrate (Sigma) was dissolved in purified water to a final concentration of 1.0 × 10^−3 ^M. Then 34.6 × 10^−3 ^M trisodium citrate (*J*.* T*.* Baker*) was quickly added to the boiling gold chloroauric acid solution under vigorous stirring.

After the color of the hydrogen tetrachloroaurate solution turned from pale yellow to purplish red, the resulting nanoparticulate suspension was removed from the heat and cooled at room temperature for at least 30 min. The gold nanoparticle solution was then centrifuged and diluted in 20 × 10^−3 ^M HEPES buffer (pH 7.4, Sigma) to a final concentration possessing an optical density of 0.8 at 532 nm, 0.24 nM in concentration according to Beer-Lambert law. The diluted solution was subsequently filtered through a Millipore filter (0.22 *μ*m) in order to ensure sterility.

### 2.2. Antibody Labelled Gold Nanospheres

Fifteen microlitres of anti-Mucin 7 monoclonal antibodies (mouse; abCAM ab55542) were diluted in 150 *μ*L of 20 × 10^−3 ^M HEPES buffer (pH 7.4), and 3 mL of nanoparticulate suspension (0.24 nM) was mixed with the diluted antibody solution at room temperature. In order to prevent aggregation, the solution was shaken for 5 minutes before 300 *μ*L of 1% polyethylene glycol (PEG, MW = 3400, Sigma) was quickly added. After incubation for half an hour, the solution was centrifuged at 4000 rpm for 15 minutes to remove unbound antibodies and PEG. Finally, the GNP pellets were redispersed in HEPES buffer (pH 7.4, Sigma) and maintained at 4°C.

In the first control group (control group 1), thio-PEG was added during the synthesis of anti-Mucin 7/Au. Thio-PEG has very high affinity to gold nanoparticles; thus, it is able to block the antibodies from linking to the GNPs. The second control group (control group 2) only underwent exposure to laser light.

### 2.3. GNP/Cell Carcinoma Incubation and Laser Therapy

The malignant urothelial cell lines MBT2 (murine), T24, 9202, and 8301 (human) were obtained from the Tri-Service General Hospital. All cell lines were grown in 37°C and maintained in a humidified atmosphere pressure of 5% CO_2_. We used Roswell Park Memorial Institute 1640 (GIBCO-BRL) medium with 10% fetal bovine serum (FBS) and 1% penicillin and streptomycin. The medium was changed three times a week.

The cells were then cultured in a 6-well tissue culture plate with seeding density of 200,000/well. Cells were cultured for 4 days and then 1 mL of antibody conjugated GNPs was added. Following incubation at 37°C for 30 min, the cells targeted by anti-Mucin 7/Au nanospheres were washed three times to remove all unbound nanoparticles.

For the laser therapy experiment, we used an IDAS Laser System (WaveLight Co.) to provide stable power with a pulse wave to prevent the medium from boiling. The laser was focused on 6-well tissue culture plate to form a round spot 5 mm in diameter on the sample. In order to promote thermal efficiency, the wavelength at 532 nm overlapped with the absorption region of the GNPs.

The cells then underwent 500 exposures to a 532 nm laser at various power densities and were then imaged using a Motic AE21 microscope at 40x magnification. Cell viability was determined using 0.4% trypan blue stain (Sigma). Cells stained blue were considered dead.

### 2.4. Confocal Spectral Microscopy

To observe the expression of Mucin 7 in cell membrane, we use confocal spectral microscopy (Leica SP2). The procedures were shown as follows. The cells were washed 3 times for 5 minutes and then submerged in 75% alcohol at 4 degrees for 20 minutes. The cells were then washed 3 times with PBS. The cells were then permeabilized and blocked with permeabilization solution (0.2 g BSA and 100 *μ*L Triton X-100 in 10 mL PBS) for 30 minutes at room temperature. Then the anti-Mucin 7 (mouse) primary antibodies 0.2 mg/mL were diluted 40 times with PBS and the plate was immersed for 30 minutes at room temperature. After washing it 3 times for 5 minutes, the sample was submerged by diluted gout secondary antibodies (2 mg/mL) 1 : 1000 with the same PBS solution in dark room at room temperature for one hour. Then, after washing it 3 more times, we added a bit of glycerin-PBS (1 : 1) solution and mounted it. The excitation wavelength is 495 nm and emission wavelength is 519 nm.

### 2.5. Flow Cytometry

In order to obtain the accurate expression of cell lines, flow cytometry (BD FACSCanto) was used for absolute quantification. At first, 2 × 10^7^ cells/mL were washed; 50 *μ*L of the cell suspension (approximately 1 × 10^6^ cells) was taken into a test tube. The cells were then incubated with diluted anti-Mucin 7 antibody (0.2 mg/mL to 40x) at 4 degrees in the darkened room for 30 minutes. In the next step, the TCC cells were centrifuged with 3 mL PBS at 1500 rpm (300 ×g) for 5 minutes to remove the unbound antibodies and subsequently stained with the 0.5 mL of fluorescein (Alex Flour-488) secondary antibody 1 : 1000 for 30 minutes at 4 degrees in the darkened room. At last, these stained cells were subjected to repeated centrifugation for 5 minutes with PBS to remove the superfluous secondary antibody. Then the remaining TCC cells were preserved in 0.5 mL cold PBS with 1% paraformaldehyde to prepare for analysis within 24 hours.

## 3. Results

### 3.1. GNP Characterization

**Figure 1:**
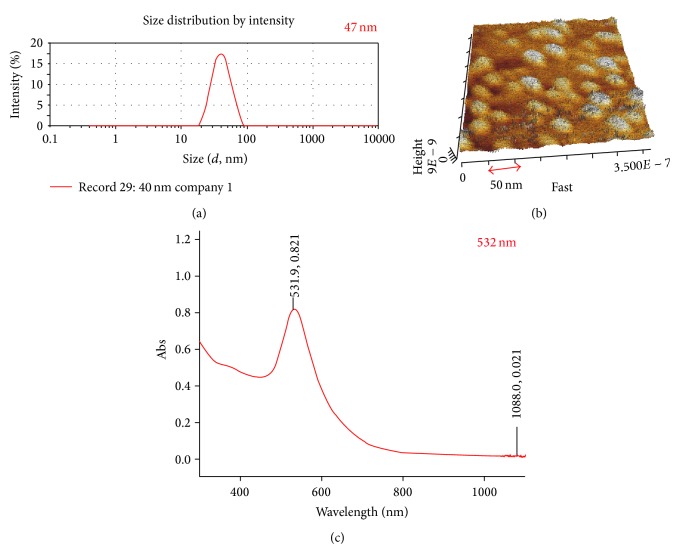
(a) DLS, (b) AFM, and (c) UV/Vis spectrophotometer images show that the average GNPs size is 47 mm, shape is round, and absorption wavelength is 532 nm.

We observed the average size and shape of gold nanoparticles using dynamic light scattering (DLS) and an atomic force microscope (AFM). The particles were spherical in shape with an average diameter of 47 nm (Figures 1(a) and 1(b)).

UV/Vis spectrophotometry showed that the absorption spectrum of GNPs was approximately 532 nm (Figure 1(c)). Thus, the wavelength of our laser system overlapped the absorption region of GNPs, thereby enhancing thermal efficiency.

### 3.2. Differential Expression Levels of Mucin 7 in the Four Cancer Cell Lines

**Figure 2:**
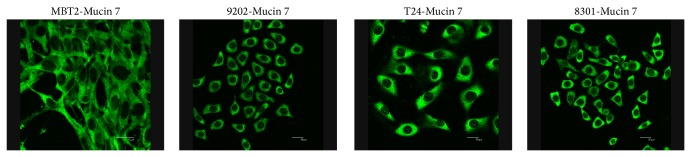
Confocal microscopic image of Mucin 7 expression in MBT2 (mice), 9202 (human), T24 (human), and 8301 (human) cancer cell lines. All cells types presented Mucin 7 on the cell membrane. The scale bar is 20 *μ*m in length.

Differential expression levels of Mucin 7 in the four cancer cell lines were observed using a confocal microscope. All cells were subjected to standard procedures of confocal microscope including fixation, cell membrane perforation, first antibody conjugation, and second antibody conjugation. The emission color of the second antibodies against Mucin 7 was green (532 nm).  Figure 2 shows that all cancer cells expressed Mucin 7 on the cell membrane.

**Figure 3:**
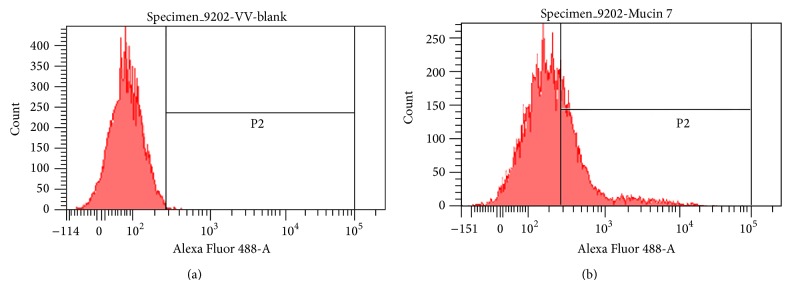
The histogram of the flow cytometry of 9202 cell line; others are similar to 9202. The right shift of the histogram of flow cytometry indicates the presentation of Mucin 7 of the cell.

We also investigated antigen expression in cell lines using flow cytometry. Our results indicated strong Mucin 7 expression (right shift) in mice (MBT2) as well as in human cancer cells (T24, 9202, and 8301).  Figure 3 shows the result of the cell line 9202.

### 3.3. Photothermal Therapy for Urothelial Cancer Cell Lines

**Figure 4:**
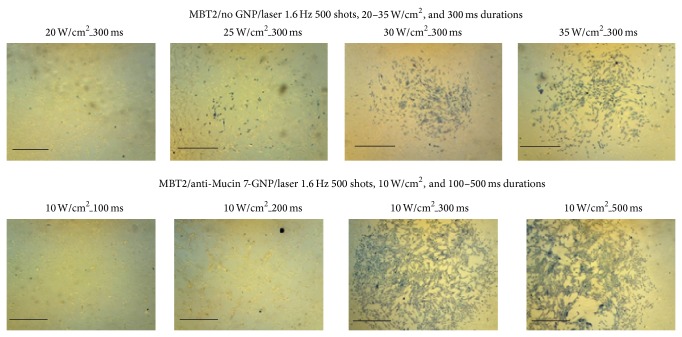
MBT2 cells treated by anti-Mucin 7 conjugated gold nanoparticles and 500 laser shots at 532 nm and a frequency of 1.6 Hz but for various durations of application. Destroying the cancer cells in the control group required more than 3 times the energy of the treatment group. The scale bar is 1 mm in length.

**Figure 5:**
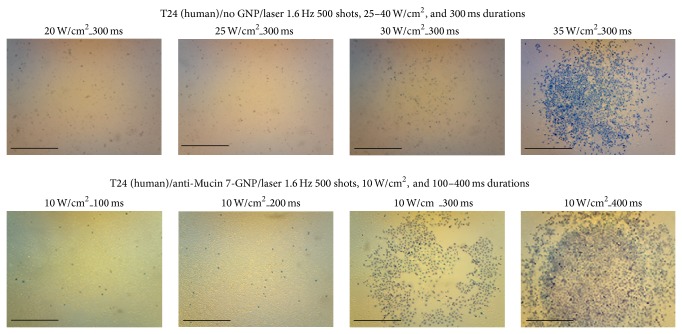
Human urothelial cancer cells (T24) treated by anti-Mucin 7 conjugated gold nanoparticles under the same conditions as those used to treat MBT2 cells. Results were similar between T24 and MBT2 cells. The scale bar is 1 mm in length.

The efficacy of PTT in the treatment of various TCC (MBT2, T24, 9202, and 8301) cell lines was tested, the results of which are presented in Figures 4 and 5. In this study, laser energy (532 nm) was applied to the urothelial cancer cell lines conjugated by anti-Mucin 7/Au nanospheres. Affected cell walls were exposed to 500 shots from a laser with a frequency of 1.6 Hz. The duration of laser shots was varied between 100 ms and 400 ms.  Figure 4 shows that MBT2 cancer cells could be killed with a 300 ms shot of approximately 10 W/cm^2^, while the nontargeted cells required 35 W/cm^2^to achieve the same effect (>0.3 cm in diameter). Our results suggest that anti-Mucin 7/Au nanospheres are able to kill cancer cells with minimum laser energy.

We were able to replicate these results in human cell lines (Figure 5), and the power level required to destroy human cancer cells was similar to that required to kill mice cancer cells. Our results demonstrate that cancer cells attacked by GNPs were damaged at a lower energy level (10 W/cm^2^, 300 ms), compared with the control group that was not treated with GNPs. All cells in the control group survived laser treatment of 10 W/cm^2^ for 300 ms.

### 3.4. Anti-Mucin 7 Antibodies Conjugated GNPs versus Anti-Mucin 7 Antibodies Blocked GNPs

**Figure 6:**
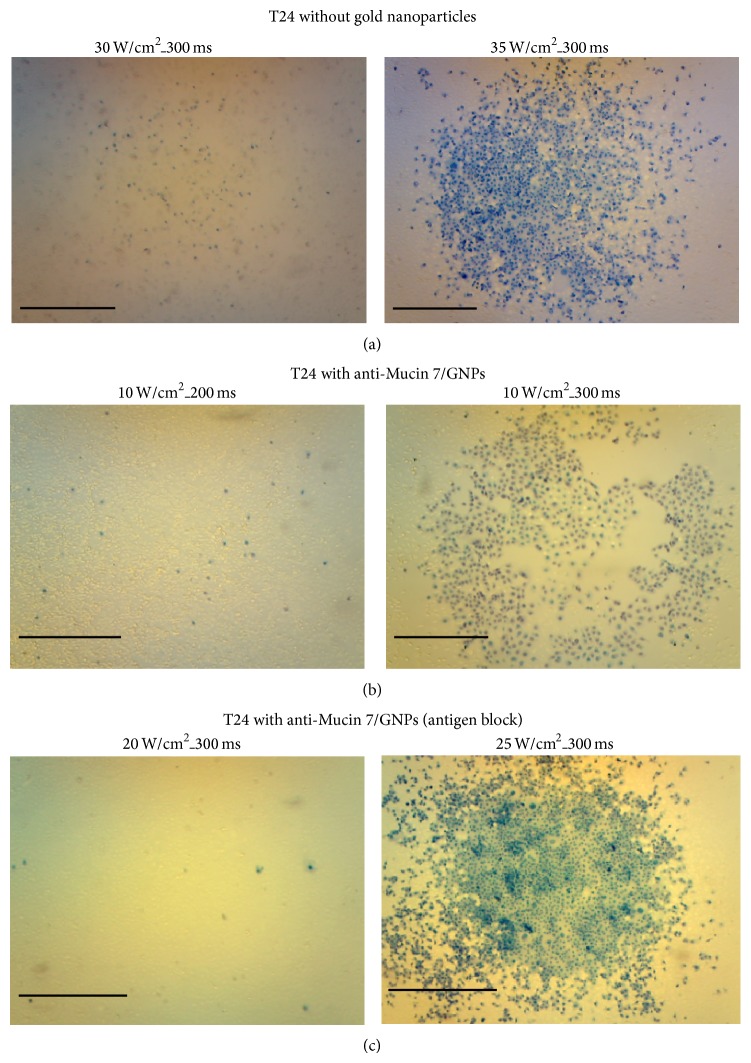
(a) T24 cells treated with laser only. Cells began to die after 500 laser shots with power of 35 W/cm^2^ 300 ms in duration at a frequency of 1.6 Hz. (b) T24 cells treated with Mucin 7 conjugated gold nanoparticles. Cells died at a power of 10 W/cm^2^ (with the same frequency and duration as described above). (c) Antibodies were blocked by thio-PEG during synthesis, and killing cancer cells required 25 W/cm^2^ of power (with the same frequency and duration as described above). The scale bar is 1 mm in length.

The urothelial cancer cells treated with Mucin 7/Au nanospheres and laser exposure were killed at a very low energy level; destroying cells in control group 1 and control group 2 required greater energy levels. Urothelial cancer cells in humans and mice began dying when exposed to similar energy levels, as shown in Figure 6.

In control group 1, the antibodies were blocked by thio-PEG and killing the cancer cells required energy levels 2.5–3.5 times higher than what was required in the experimental group. Killing cancer cells in control group 2 that received laser treatment only required energy levels 3–3.5 times higher than that required in the experimental group. More specifically, achieving similar killing efficiency in the experiment group required an energy level of only 10 W/cm^2^. Energy levels below this left nearly all cells alive.

## 4. Discussion

Safety and effectiveness are key issues in medical laser applications. One of the advantages of treatment with gold nanoparticles is the photothermal properties, which can convert absorbed photons into thermal energy. This process is facilitated by the oscillation of free electrons on the surface of the gold nanoparticles, and the laser energy required to achieve this transformation is far below those stipulated in medical safety standards. Consequently, determining the means to fabricate a functionalized nanoparticle is necessary for the effective targeting of tumor cells. Efficient targeting can improve the efficacy of treatment and reduce nonspecific bonding of gold nanoparticles to benign cells.

We determined the expression level of Mucin 7 in four cell lines using confocal microscopy and flow cytometry. Results from this analysis clearly confirmed the presence of this protein in cancer cell lines. Based on these findings, we selected gold nanospheres to conjugate with anti-Mucin 7 antibodies for photothermal therapy.

Immunized photothermal therapy refers to a combination of target therapy and photothermal therapy. In this treatment technique, antibodies comprise the targeting system and gold nanoparticles destroy the cell membrane of targeted cells when exposed to light at a selected wavelength.

Thio-PEG has a very high affinity to gold nanoparticles, which makes it possible for thio-PEG to block antibodies from binding to immunized gold nanoparticles during the synthesis process. If the antibodies are unable to conjugate the GNPs, the GNPs will lose the ability to target the cancer cells. We observed the compatible conditions in our study; the control group with thio-PEG/GNPs required similar laser energy comparing to that required in the control group without GNPs.

Antibodies conjugated GNPs and thio-PEG-GNPs can prevent the aggregation and deposition because they form a membrane layer outside the GNPs. For bare GNPs, the experiment is hardly reproduced because the GNPs will aggregate and deposit when they contact the culture medium and cells due to the nonspecific conjugation. After washing, the condition differs all the time.

Urothelial cancer, which is the most common type of urinary bladder cancers, usually requires transurethral resection. Approximately 70% of urothelial cancers are initially diagnosed as Ta-T1, which are nonmuscle invasive. However, urothelial cancer tumors have high recurrence rates involving approximately 70% of patients experiencing tumor recurrence within the first 5 years. To delay recurrence, physicians usually apply an intravesical instillation of mitomycin or BCG. However, although BCG and mitomycin instillations have been proven to delay recurrence, they cannot prevent it indefinitely, implying that this approach is unable to kill undetectable tumors.

In this study, we found that killing cancer cells in the treatment group required only one third of the energy required for the control group. However, using different sized culture plates may yield different results because culture media play a cooling role during PTT. Thus, using smaller plates could lead to more cell death. In this study, we used 6 well plates because the results from a culture plate of this size are similar to those obtained using bigger plates. In addition, the culture medium was not heated above 40°C during the experiments, thereby ensuring that cells were damaged by PTT rather than being boiled in the medium.

Findings from this study can be applied in various stages of treatment for nonmuscle invasive urothelial cancer. For example, if a patient has already received a standard TURBT procedure, PTT can be used to kill any residual tumors. In many cases where nonmuscle invasive urothelial cancer cells are invisible to cystoscopy, PTT may be the optimal treatment approach. PTT effectively targets and kills residual cancer cells and also preserves healthy muscle layer. Once the muscle layer acquires widespread fibrosis, it loses both contractility and compliance, resulting in degraded quality of life and potential renal failure. Selecting a 532 nm green light laser for PTT can maximize the benefits and reduce the harm, due to the fact that penetration depth of the laser is shallow at this wavelength.

In urothelial bladder cancers, intravesical instillation of nanoparticles and intravesical exposure of laser energy can considerably lower the chance of depositing the nanoparticles in healthy tissue because they can be immediately voided after treatment; however, this issue requires verification in animal studies. This characteristic makes PTT a plausible treatment-course for human subjects with the minimal risk of toxicity to healthy organs, such as the liver, lung, brain, and kidney.

Nonetheless, there are limitations to our present research. For example, we only conducted an in vitro study under restricted conditions and the in vivo dosages required to kill cancer cells may be different. Thus, further studies on animals and humans are required to determine the correct dosage of GNPs and the appropriate laser exposure when this treatment is applied in vivo.

## 5. Conclusions

Photothermal therapy using immunized gold nanospheres was shown to significantly reduce the level of energy required to kill targeted cancer cells compared with the nontargeted cells in the control groups. This technique is also able to avoid damage to normal cells. Photothermal therapy with immunized gold nanospheres shows considerable promise in the treatment of urothelial cancer; however, further study using animal models is required to confirm our results.
